# Venous Thromboembolism in Patients With Brain Cancer: Focus on Prophylaxis and Management

**DOI:** 10.7759/cureus.8771

**Published:** 2020-06-22

**Authors:** Sohaip Kabashneh, Samer Alkassis, Layla Shanah, Ala A Alkofahi

**Affiliations:** 1 Internal Medicine, Wayne State University/Detroit Medical Center, Detroit, USA; 2 Internal Medicine, University of Hawaii, Honolulu, USA

**Keywords:** venous thromboembolism (vte), brain tumors cns tumors, prophylaxis, intracranial hemorrhage, neurosurgery

## Abstract

Patients with primary and metastatic brain tumors are predisposed to thromboembolism. This review of the literature explores the high prevalence of venous thromboembolism and its negative impact on patients with brain cancer. It outlines the recommended prophylactic strategies to prevent venous thrombosis and analyzes the benefit versus risk of anticoagulation in this population, with a focus on the risk of intracranial bleeding associated with it. Additionally, it explores the exceedingly high prevalence of venous thromboembolism in the setting of brain cancer surgeries and provides guidance on the best methods used for prophylaxis in this setting and discusses the safety of each method perioperatively. Lastly, this review article provides guidance on how to manage venous thromboembolism in patients with brain cancer and discusses the use of vena cava filters in this population.

## Introduction and background

Brain tumors represent a diverse group of neoplasms classified into either primary brain tumors, which originate from within the central nervous system (CNS), or secondary brain tumors (metastatic), which are spread from a tumor elsewhere in the body. Cancer patients in general are at significantly increased risk of developing venous thromboembolism (VTE) [1,2]. However, patients with brain tumors are at even higher risk of VTE, possibly because brain tumors inhibit plasmin, enhance the release of thromboplastin, and increase procoagulant and platelet aggregation activity [3-6].

Deep vein thrombosis (DVT) and subsequent pulmonary embolism (PE) are associated with significant morbidity and mortality in oncology patients. VTE occurs in approximately 10% of patients, making it the second leading cause of mortality in cancer patients, second only to death from cancer itself [7-9]. In fact, patients who experience VTE will have a lower quality of life and survival rate than those who do not. Studies found a 30% increase in two-year mortality in patients with malignant glioma who develop VTE [6]. 

As a result of the profound negative impact of thromboembolism in patients with brain tumors, DVT prophylaxis seems necessary [10,11]. However, the risk of intracranial bleeding makes the decision to use anticoagulation challenging as some types of brain tumors have a high propensity to bleed spontaneously [12]. Intracranial bleeding is a significant complication that requires discontinuation of anticoagulation in this population.

In this review of the literature, we aim to shed light on the prevalence and significance of VTE in patients with brain tumors. In addition, we will determine whether DVT prophylaxis is reasonable and safe in this population, particularly in the perioperative setting. Lastly, we will discuss the management guidelines of VTE in this population.

## Review

Brain cancers impose a high risk of thrombotic events compared to other cancer types

According to prospective data, having cancer is associated with a 4.1-fold increased risk of VTE compared to the general population. In patients undergoing chemotherapy, the risk increases 6.5-fold [1]. Furthermore, evidence suggests that the absolute risk depends on the tumor type and the stage or extent of cancer [13].

Patients with primary or metastatic brain tumors are more prone to develop VTE compared to patients with other types of cancers. The incidence rate of VTE is 20%-30% in high-grade glioma and up to 20% in metastatic brain disease [6]. Horsted et al. studied the association between VTE and various cancer types. Cancers of the pancreas and brain were associated with the greatest and second greatest risk of VTE, respectively [14].

Cancer treatments are also associated with an increased risk of thrombosis. Lee and Levine found very high rates of VTEs in patients treated with combination therapy including an antiangiogenic agent [13].

The use of long-term prophylactic anticoagulation in brain tumor outpatients 

Perry et al. conducted a small clinical trial to evaluate the safety of tinzaparin for DVT prophylaxis in newly diagnosed grade III-IV malignant glioma patients. A total of 40 patients were started on tinzaparin for a median time of 161 days. Two patients developed brain hemorrhages: one patient had grade I and another patient had grade II. There were no patients with a grade IV or V brain hemorrhage or systemic hemorrhage greater than grade II. The overall incidence of minor brain hemorrhage in the study was 5% compared to 3% chance of spontaneous brain hemorrhage. No major brain bleed occurred. The study concluded that tinzaparin at a fixed prophylactic dose is safe in brain tumor patients [15].

The PRODIGE is the largest randomized trial to assess the use of anticoagulation for VTE prophylaxis in brain cancer patients to date. A total of 186 patients with newly diagnosed malignant glioma were randomized to either dalteparin (5,000 IU) or placebo for a total of 12 months. Unfortunately, the study was terminated prematurely because of the expiration of the study medication. This study suggested that there were reduced episodes of VTE and increased intracranial bleeding in the dalteparin thromboprophylaxis group. However, neither finding was statistically significant to draw definitive conclusions [16].

Overall, given the literature available to date, the use of prophylactic or even therapeutic anticoagulation does not seem to increase the risk of major intracranial bleeding in patients with brain cancer. However, there is no consistent evidence to dictate the use of long-term primary pharmacological VTE prophylaxis in this group. In fact, the International Society on Thrombosis and Haemostasis (ISTH) recommends against routine thromboprophylaxis in cancer outpatients, especially without risk assessment [17].

VTE risk and prophylaxis in brain cancer surgeries

High-grade gliomas carry one of the highest risks of perioperative VTE. In fact, when comparing craniotomy for a brain tumor with craniotomy for non-neoplastic disease, rates of postoperative VTEs are twice as high [18,19]. VTE occurs in up to 21% in the first three months after craniotomy for brain tumors [20,21]. Additionally, Chan et al. found that VTE rates were higher for patients who underwent surgery for metastatic brain tumors compared to those who underwent surgery for primary brain tumors [22].

Senders et al. collected data from the National Surgical Quality Improvement Program (NSQIP) registry (2005-2015) for all patients who underwent craniotomy for a primary malignant brain tumor. Among the 7,376 patients who were identified, VTE was the second most common major complication occurring in 257 (3.5%) patients. Notably, only 36% of VTEs occurred during the hospital stay. Intracranial hemorrhage (ICH) was the most common reason for reoperation in this study population. Approximately 1.3% developed an ICH requiring surgical evacuation at a median of two days after surgery. These findings suggest an increased risk of VTE beyond the period of hospitalization, whereas ICH occurs predominantly within the first few days after surgery [23].

Due to the prevalence of VTE and its significance in brain cancer surgeries, VTE prophylaxis in this setting seems to be warranted. However, this must be carefully weighed against the risk of ICH. In an attempt to weigh risk vs benefit of VTE prophylaxis, Constantini et al. conducted a randomized trial on 103 patients undergoing brain tumor surgery. A total of 55 patients were given 5,000 units of unfractionated heparin (UFH) every 12 hours and 48 patients were given placebo starting two hours before surgery and continuing until full mobilization or for seven days. The study revealed that prophylactic heparin did not increase the risk of bleeding and it is safe to be administered perioperatively in craniotomy [24]. Authors of other studies reported similar results, prophylactic heparin administered perioperatively for neurosurgical patients is safe to use. However, these studies focused on the general population of neurosurgery patients and were not restricted to patients with brain tumors undergoing craniotomy [25,26].

In order to identify what prophylactic modalities provide the most protection against VTE in neurosurgical patients, Agnelli et al. compared a combination of enoxaparin and compression stockings with compression stockings alone for VTE prophylaxis after neurosurgery. The study concluded that enoxaparin combined with compression stockings is more effective than compression stockings alone for the prevention of VTE and does not cause excessive bleeding [27]. Nurmohamed et al. also concluded that a combination of anticoagulation and compression stockings decreases the risk of VTE significantly without causing any significant increase in major bleeding [28]. The American College of Chest Physicians (ACCP) also recommends both pharmacologic prophylaxis and mechanical prophylaxis for patients with brain tumors undergoing surgery [29]. Overall, there is a wide agreement in the literature and societies to use both pharmacologic prophylaxis and mechanical prophylaxis in patients with brain tumors undergoing surgery, which is both effective and safe.

VTE management in patients with brain cancer 

In the past, patients with brain tumors and VTE were often managed with the placement of inferior vena cava (IVC) filters rather than with anticoagulation due to concern of brain hemorrhage [30]. However, a retrospective cohort study of 293 cancer patients with brain metastases by Donato et al. showed no differences in the one-year cumulative incidence of significant ICH in patients who received therapeutic doses of enoxaparin compared with those who did not [31].

The most updated American Society of Clinical Oncology (ASCO) practice guidelines recommend anticoagulation for patients with primary or metastatic brain malignancies and an established VTE. This is based on a large systematic review that included a total of 35 publications (26 meta-analyses and nine randomized controlled trials) on VTE conducted by Key et al. [32]. The guidelines also discussed that initial anticoagulation may involve low-molecular-weight heparin (LMWH), UFH, fondaparinux, or rivaroxaban. For patients initiating treatment with parenteral anticoagulation, LMWH is preferred over UFH for the initial five to ten days of anticoagulation for newly diagnosed VTE in the absence of severe renal impairment [32].

All the recommendations regarding the insertion of a vena cava filter were made with informal consensus and low-quality evidence. It may be offered to patients with absolute contraindications to anticoagulant therapy in the acute treatment setting if the thrombus burden is considered life-threatening [32].

## Conclusions

VTE is a common problem in patients with brain cancer and has a significant negative impact on this population. The use of prophylactic anticoagulation in the outpatient setting is not recommended. Brain cancer surgery is a high risk for thromboembolism; therefore, a combination of both pharmacologic and mechanical prophylaxis is recommended perioperatively. Therapeutic anticoagulation is safe and effective in this population is. Strong evidence for the utilization of a vena cava filter in this population is lacking.
